# Radiofrequency catheter ablation-associated silent iatrogenic right ventricular pseudoaneurysm: a case report and literature review

**DOI:** 10.3389/fcvm.2025.1631315

**Published:** 2025-10-07

**Authors:** Shogo Haruki, Hiroyuki Yamamoto

**Affiliations:** ^1^ Department of Cardiology, Chiba-Nishi General Hospital, Chiba, Japan; ^2^ Department of Cardiology, Tokyo Medical University Hospital, Tokyo, Japan

**Keywords:** right ventricular pseudoaneurysm, radiofrequency catheter ablation, ventricular outpouching, cardiac computed tomography angiography, iatrogenic

## Abstract

**Background:**

Right ventricular pseudoaneurysm (RVP) encased by adjacent pericardial or scar tissue is rare but can be a fatal sequela of cardiac rupture. Differentiating between pseudoaneurysms and true aneurysms is important because they have different natural histories and require distinct treatments. Radiofrequency catheter ablation (RFCA) is a potential cause of RVP; however, RFCA-associated RVP incidence and management remain unclear.

**Case presentation:**

An 88-year-old woman with refractory paroxysmal supraventricular tachycardia was admitted to our hospital. We performed an electrophysiological study, which led to a final diagnosis of atrioventricular nodal reentrant tachycardia, for which successful RFCA was performed. On post-procedural day 2, echocardiography revealed a small right ventricular apical outpouching. Cardiac computed tomography angiography led to the correct diagnosis of RVP, which was successfully treated with surgical repair. The postoperative course was uneventful.

**Conclusions:**

We describe a unique case of RFCA-associated apical RVP. This case highlights the importance of the potential risk of iatrogenic RVP and the value of cardiac computed tomography angiography in diagnosing RVP in patients with right ventricular outpouching.

## Highlights

• Right ventricular pseudoaneurysm (RVP) is potentially fatal if left untreated.
• Silent iatrogenic RVP may develop following radiofrequency catheter ablation.
• Cardiac computed tomography angiography is valuable for diagnosis of RVP.

## 1 Introduction

Ventricular pseudoaneurysms may occur in association with myocardial infarction, trauma, infection, catheter-related procedures, cardiac surgery, or idiopathic (1). Cardiac pseudoaneurysms often occur in the left ventricle, whereas right ventricular pseudoaneurysms (RVP) are rare (2). We describe a unique case of silent iatrogenic RVP secondary to radiofrequency catheter ablation (RFCA).

## 2 Case description

**Figure 1:**
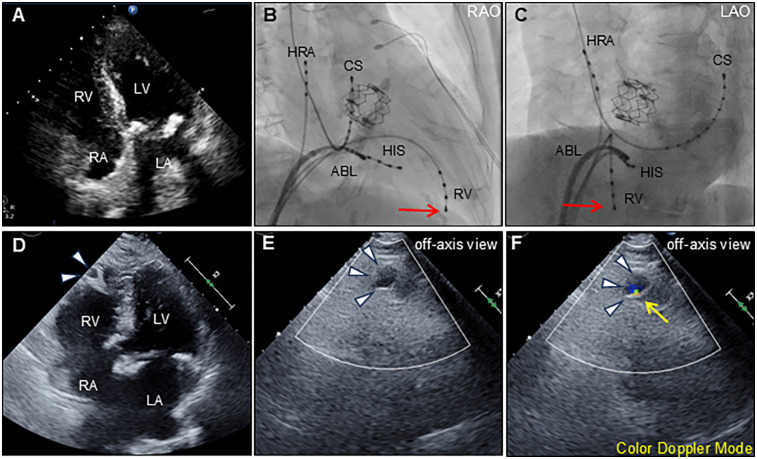
Transthoracic echocardiography and ablation images. Preprocedural transthoracic echocardiography (apical 4-chamber view) shows normal biventricular function with normal anatomy **(A)** Radiographs of the right anterior oblique [RAO; **(B)**] and left anterior oblique [LAO; **(C)**] views show the radiofrequency ablation catheter (ABL) positioned at the posterior right atrial septum. Other catheters were positioned at the high right atrium (HRA), His-bundle (HIS), coronary sinus (CS), and right ventricle (RV). Red arrows indicate the tip of the indwelling RV catheter. Postprocedural transthoracic echocardiography (apical 4-chamber view) reveals an outpouching of the right ventricular apex (arrowheads) **(D)** Color Doppler echocardiography using an off-axis view reveals outpouching of the right ventricular apex (arrowheads) during diastole **(E)** and systole **(F)** The expansion with the antegrade flow at systole, suggests communication between the outpouching and right ventricle (yellow arrow). LA, left atrium; LV, left ventricle; RA, right atrium; RV, right ventricle.

**Figure 2:**
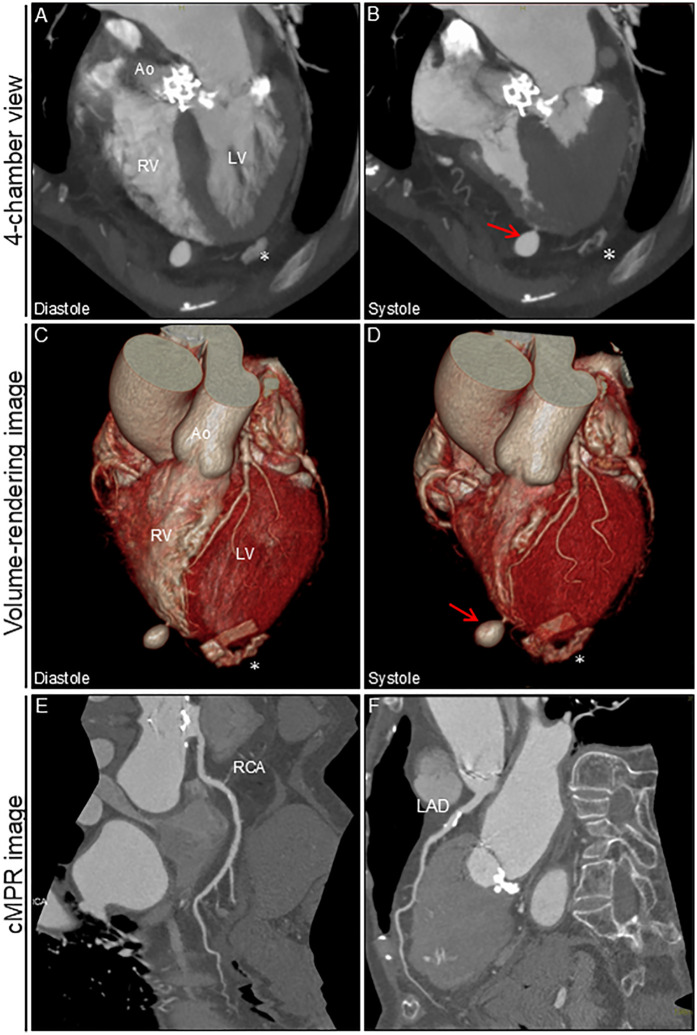
Cardiac computed tomography angiography image after ablation. 4-chamber view and volume-rendering images during diastole **(A,C)** and systole **(B,D)**. Outpouching with a narrow neck at the apex of the right ventricle is markedly prominent during systole (arrows). No significant pericardial effusion was observed. cMPR images of RCA **(E)** and LAD **(F)** * Denotes the remnants of felt in the left ventricular apex after transapical aortic valve replacement. Ao, aorta; cMPR, curved multi-planar reformation; LAD, left anterior descending artery; LV, left ventricle; RCA, right coronary artery; RV, right ventricle.

**Figure 3:**
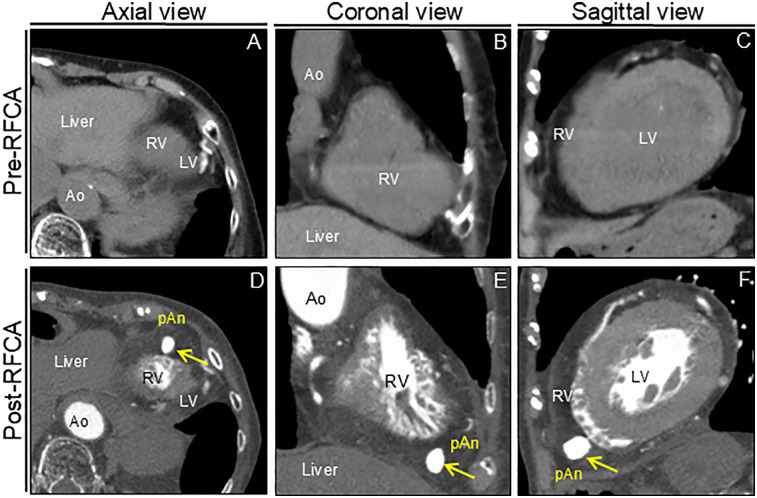
Computed tomography images before and after RFCA. Preprocedural unenhanced computed tomography images confirm normal right ventricular anatomy **(A–C)**. Postprocedural cardiac computed tomography angiography images reveal apical outpouching of the right ventricle (arrows) **(D–F)**. Ao, aorta; LV, left ventricle; pAn; pseudoaneurysm; RFCA; radiofrequency catheter ablation; RV, right ventricle.

An 88-year-old woman was admitted with symptomatic paroxysmal supraventricular tachycardia that had persisted for three years. The patient had a history of severe aortic stenosis for which transapical transcatheter aortic valve replacement was performed seven years ago. Her initial vital signs were blood pressure of 79/60 mmHg and heart rate of 164 beats/min. The physical examination findings were unremarkable. Laboratory test revealed elevated serum level of brain natriuretic peptide (171.6 pg/ml, reference: <18.4 pg/ml). Electrocardiography revealed narrow QRS tachycardia with a short RP (Supplementary Figure S1A). Tachycardia was terminated with rapid intravenous administration of adenosine triphosphate (Supplementary Figure S1B). However, the patient experienced frequent paraoxymal supraventricular tachycardia episodes. Echocardiography revealed no structural or functional heart abnormalities (Figure 1A; Supplementary Video S1). Unenhanced computed tomography scans showed no biventricular abnormalities suggestive of myocardial infarction or aneurysm. An electrophysiological study was performed four days after admission. Catheters were placed in the high right atrium, the His bundle region, coronary sinus, and right ventricular (RV) apex. Premature ventricular contractions frequently occurred during RFCA, and, hence, the RV pacing catheter was held tightly to avoid the unintended catheter movement. Based on the electrophysiological study, slow/fast atrioventricular nodal reentrant tachycardia was diagnosed. Subsequent successful anatomical slow-pathway ablation was performed according to standard techniques (Figures 1B,C). We excluded any complications, including cardiac tamponade, on postprocedural echocardiography. Follow-up echocardiography revealed RV apical outpouching on postprocedural day 2 (Figures 1D–F; Supplementary Video S2). The patient was asymptomatic, and her vital signs were stable. Physical examination and electrocardiographic findings were unremarkable (Supplementary Figures S1C,D). Follow-up laboratory tests were close to normal. Differential diagnoses of ventricular outpouching include true aneurysms and pseudoaneurysms. Cardiac computed tomography angiography (CCTA) further characterized the morphology and features of the RV apical outpouching (Figures 2A–D). Note the presence of contrast-filled RV outpouching at the apex that protruded during systole, with a maximum diameter of 12.1 mm and a narrow orifice of 1.5 mm with an orifice-to-maximum diameter ratio of 12.4%, suggestive of RVP. CCTA revealed normal coronary arteries (Figures 2E,F). Pericardial effusion was not observed. A detailed review of the computed tomography images confirmed the absence of RVP before the RFCA procedure and its presence after the procedure (Figure 3). Given the temporal relationship between RFCA and the occurrence of RVP without any other identifiable cause, a final diagnosis of iatrogenic RVP was made. After multidisciplinary discussion, taking into consideration that a ventricular pseudoaneurysm is susceptible to cardiac rupture, the patient underwent urgent surgical repair of the RVP. No bleeding was observed in the pericardial sacs. There was no evidence of pericarditis, intrapericardial bleeding, or cardiac rupture except for a slight bulge at the RV apex. Epicardial echocardiography was used to identify the pseudoaneurysm, as such pseudoaneurysm was difficult to identify by visual examination. Subsequent vertical mattress suture repair with Teflon-felt reinforcement was performed for the RVP. The patient's postoperative course was uneventful, and she remained asymptomatic at the one-year follow-up (Supplementary Figure S2).

## 3 Discussion

Here, we describe a unique case of silent iatrogenic RVP after RFCA. Our case provides the following clinical lessons: We reviewed the previously reported cases of RFCA-associated cardiac aneurysms (3–25), which were published in English in PubMed between 1985 and 2024 (Table 1). The left ventricle was the most common site of origin of the cardiac aneurysm, followed by the right ventricle, mitral-aortic intervalvular fibrosa, and right atrium. As in previous studies (26), nearly 30% of the patients had no clinical symptoms. The literature documents the triggers, characteristics, management, and outcomes of RVP after RFCA in two cases (6, 11). Notably, RVP developed at a site far from the ablation target site in the present case. Given the histological nature of the RV, which is composed of fragile pectinate muscles with wall thinning, age-related RV myocardial degeneration, physical stress on the RV myocardium due to prior temporary pacing wire placement and improper manipulation of the RV catheter tip might have triggered the RVP. Thus, for medical specialists in RFCA, recognizing these rare RFCA-related complications may aid in early detection and proper management. CCTA with good temporal and spatial resolutions can clearly differentiate ventricular outpouchings (27). Ventricular pseudoaneurysms are characterized by a narrow opening that connects to the cardiac chamber. Pseudoaneurysms expand outward in response to the increased intraventricular pressure during systole. Indeed, CCTA was valuable for the accurate diagnosis of silent iatrogenic RVP in the present case. There are currently no guidelines for the appropriate management of ventricular pseudoaneurysms. Given the high risk of cardiac rupture, surgical repair has been considered the first-line treatment of choice for ventricular pseudoaneurysms (1). Although surgical repair was performed, there was no evidence of impending cardiac rupture in the present case. Considering the high surgical mortality rate of 7%–23% (28), conservative management to reduce the risk of cardiac rupture might be another treatment option (26, 29). Percutaneous embolization with coils offers another promising therapeutic alternative, limited to midterm outcomes (29, 30). Owing to the nature of our hospital, which did not have an experienced interventionalist but had many experienced cardiac surgeons on staff, we decided to opt for surgical treatment in this case. Moreover, in our review, nearly half the patients received conservative management with a favorable prognosis. Noninvasive treatment strategies may be feasible in elderly patients with stable hemodynamics and no risk of cardiac rupture. Data on the long-term outcomes of the above treatments is lacking, and future evidence is awaited to be built up through data accumulation.

**Table 1:** Literature review of cases of cardiac aneurysm associated with radiofrequency catheter ablation.

Case	Author	Age/Sex	Underlying disease	Trigger factor	Cardiac aneurysm							Management	Outcome
		(y)			Type	Anatomy	Location	Size (mm)	PE	Clinical manifestation	Time-to-onset		
1	Salazer et al. (3)	31/M	WPW	Lateral free wall accessory pathway	P	LV	Left lateral atrioventricular groove near the coronary sinus	20	Absence	Cardiac murmur	PPD 3 months	SR	Survival
2	Mabo et al. (4)	49/M	WPW	Posterior accessory pathway	P	LV	Posterior mitral annuls	NA	Absence	None	PPD 2 months	SR	Survival
3	Gill et al. (5)	69/F	WPW	Concealed Kent posterior submitral	P	LV	Submitral in the posterolateral region of LV	NA	Absence	None	PPD 2–3 days	Con	Complete resolution at 1-month follow-up
4	Wolf et al. (6)	49/F	WPW/VT	RFCA for VT	P	RV	RV outflow tract	10 × 10 × 10	Presence	Sudden cardiac death	PPD 9 months	CPR	Died
5	Benezet-Mazuecos et al. (7)	NA	OMI	RFCA for OMI-related VT	T	LV	Apex	NA	Absence	NA	PPD 2 days	Med	Survival
6	Mansour et al. (8)	50/M	WPW	Left lateral bidirectional accessory pathway	P	LV	Basal lateral wall under the mitral annulus	30 × 40	Absence	Syncope	PPD 15 years	SR	Survival
7	Miura et al. (9)	57/M	WPW	RFCA for WPW	P	LV	Posterolateral mitral annuls	15 × 15	Presence	Cardiogenic shock	During RFCA	SR	Survival
8	Han et al. (10)	27/M	AF	RFCA for AF	P	MAIVF	MAIVF	NA	Absence	None	PPD 1 month	Con	No change at 4-year follow-up
9	Koruth et al. (11)	63/M	VT	Epicardiac puncture	P	RV	Mid-RV free wall	13 × 10 × 9	Absence	CP	After procedure	Con	Complete resolution after few weeks
10	Koch et al. (12)	60s/F	VT	RFCA for VT	P	LV	Inferior wall extending into posterolateral wall	90	Presence	ICD inappropriate shock	PPD 8 months	SR	Survival
11	Han et al. (13)	NA	AF	RFCA for AF	P	NA	NA	NA	Absence	None	PPD 1 month	NA	Partial resolution
12	Auriau et al. (14)	Young/F	WPW	Left posterolateral accessory atrioventricular pathway	P	LV	Lateral wall near the circumflex artery and the mitral annulus	37 × 44	Absence	Palpitation, faintness and dyspnea	PPD 12 years	SR	Survival
13	Dandamudi et al. (15)	52/F	PVC	RFCA for LV summit PVC	P	LV	Basal anterior wall	24 × 24	Presence	Cardiogenic shock	PPD 5 weeks	SR	Survival at 5-month follow-up
14	Kim and Lee (16)	39/M	WPW	RFCA for WPW	P	LV	Posterior mitral annuls	27 × 17	Presence	Syncope and CP	PPD 12 days	Con	No change at 1-year follow-up
15	Wang et al. (17)	69/M	PVC	RFCA for PVC	P	LV	Inferior wall near the mitral annulus along the left atrium	90 × 40	Presence	Dizziness and CP	PPD 2 days	SR	Survival
16	Watanabe et al. (18)	60/M	VT	RFCA for VT	P	LV	Left ventricular outflow tract	NA	Absence	CP	During RFCA	Med	Improved
17	Fritz et al. (19)	59/M	PVC	RFCA for PVC	P	LV	Basal inferior wall	NA	Presence	Syncope and dyspnea	During RFCA	SR	Survival
18	Nicolazzi et al. (20)	38/F	VT	Fascicular VT target to posterior lateral papillary muscle	P	LV	Mid inferior wall	20 × 14	Absence	Fatigue, palpitations, GI symptoms	PPD 1 month	SR	Survival
19	Kim et al. (21)	74/F	VT	RFCA for VT from posteromedial papillary muscle	P	LV	Posteromedial papillary muscle	26 × 9	Absence	None	PPD 2 months	Con	No change at 1-year follow-up
20	Kasai et al. (22)	82/M	PVC	RFCA for PVC	P	LV	Posterior papillary muscle	12 × 11	Absence	None	PPD 1 month	Con	No change at 3-month follow-up
21	Korkmaz et al. (23)	69/M	PVC	RFCA for PVC	T	LV	Basal middle septum	NA	Absence	VT	PPD 1 year	Con	Survival
22	Izekor et al. (24)	72/M	PVC	RFCA for PVC	P	LV	Anterolateral wall	19.5	Absence	Palpitation	PPD 34 days	SR	Survival
23	Manongi et al. (25)	80s/F	PAF	Coronary sinus catheter	P	RA	RA appendage	NA	Presence	None	PPD 2 days	Con	Symptom resolution at 3-month follow-up
24	Present case	88/F	SVT	RV catheter	P	RV	Apex	9.6 × 12.1	Absence	None	PPD 3 days	SR	Survival

AF, atrial fibrillation; CP, chest pain; Con, conservative treatment; F, female; GI, gastrointestinal; ICD, implantable cardioverter defibrillator; M, male; MAIVF, mitral-aortic intervalvular fibrosa; Med, medical treatment; NA, not applicable; OMI, old myocardial infarction; PAF, paroxysmal atrial fibrillation; PE, pericardial effusion; PPD, postprocedural day; PVC, premature ventricular contraction; P, pseudoaneurysm; LV, left ventricle; RA, right atrium; RFCA, radiofrequency catheter ablation; RV, right ventricle; SVT, supraventricular tachycardia; SR, surgical repair; T, true aneurysm; VT, ventricular tachycardia; WPW, Wolff-Parkinson-White syndrome.

Iatrogenic cardiac pseudoaneurysm have been reported in various clinical reports. Left ventricular pseudoaneurysms (LVPs) are the most frequent type of iatrogenic cardiac pseudoaneurysm, with approximately one-third arising from surgical procedures involving mitral valve replacement (1). Percutaneous device interventions predominantly including transapical transcatheter aortic valve replacement can often cause iatrogenic LVP (31, 32). Cases of transcatheter mitral valve implantation and ventricular septal defect closure device-related LVP (33, 34) have also been reported. Although iatrogenic RVPs are very rare, various case reports describe them in connection with endomyocardial biopsy (35), lead extraction (36), pericardiocentesis (30), Swan-Ganz catheter (37), valve in valve treatment of tricuspid valves (38), placement of a hemodialysis catheter (39), surgery for atrio-ventricular septal defect with tetralogy of Fallot (40) and insertion of central venous line (41). Considering the above diverse case reports, our case highlights that RVP can complicate even a routine procedure.

## 4 Limitation

Our case was finally diagnosed as iatrogenic RVP based on the temporal relationship between RFCA and evidence of *de novo* RVP, but the direct causal relationship and detailed mechanism remain unclear. In addition, histological analysis could not be performed in this case. Early onset and the absence of reactive pericardial effusion observed in our case suggests that it may have been a specific subtype of RVP with residual cardiomyocytes that are vulnerable but have not yet ruptured. Future systematic and comprehensive pathological analyses of RFCA-associated ventricular aneurysms are warranted.

## 5 Conclusions

This is a rare case of silent iatrogenic RVP after RFCA. This case report highlights the importance of recognizing iatrogenic RVP and the clinical significance of CCTA for diagnosis of RVP. Therefore, clinicians should be aware of RVP as a possible complication after RFCA and understand the imaging techniques useful for its early diagnosis and determine appropriate treatment options.

## Data Availability

The original contributions presented in the study are included in the article/Supplementary Material, further inquiries can be directed to the corresponding author.

## Supplementary material

Supplementary Figure S1 Time-course of electrocardiographic change. Electrocardiography (ECG) on emergency arrival **(A)**, after intravenous adenosine triphosphate **(ATP)** administration **(B)**, before the ablation **(ABL) (C)**, and after **the ABL (D)**. The episode was a narrow QRS with short RP tachycardia. No ST-T changes were observed before or after the ABL. The ECG paper was set to a standard speed of 25 mm/s and voltage (amplitude) of 10 mm/mV.

Supplementary Figure S2 Timeline of the diagnostics, therapeutic interventions, and disease status of the present case. AS, aortic valve stenosis; ATP, adenosine triphosphate; AVNRT, atrioventricular nodal reentrant tachycardia; BP, blood pressure; CCTA, coronary computer tomography angiography; CT, computer tomography; ECG, electrocardiography; EF, ejection fraction; EPS, electrophysiological study; HR, heart rate; PVC, premature ventricular contraction; PSVT, paroxysmal supraventricular tachycardia; RFCA, radiofrequency catheter ablation; RV, right ventricular; RVP, right ventricular pseudoaneurysm; TA-TAVR, transapical transcatheter aortic valve replacement; and TTE, transthoracic echocardiography.

Supplementary Video S1 Transthoracic echocardiography before ablation. Preprocedural transthoracic echocardiography (apical 4-chamber view) shows normal biventricular function with normal anatomy.

Supplementary Video S2 Transthoracic echocardiography after ablation. Postprocedural transthoracic echocardiography (an off-axis view) shows right ventricular pseudoaneurysm.
