# Supplementary figures and images for: Radiofrequency catheter ablation-associated silent iatrogenic right ventricular pseudoaneurysm: a case report and literature review
Source: Front Cardiovasc Med. 2025 Oct 7;12:1631315. doi: 10.3389/fcvm.2025.1631315 (PMC12537891; doi:10.3389/fcvm.2025.1631315)

**(A) On admission (B) After ATP infusion (C) Before ABL (D) After ABL**

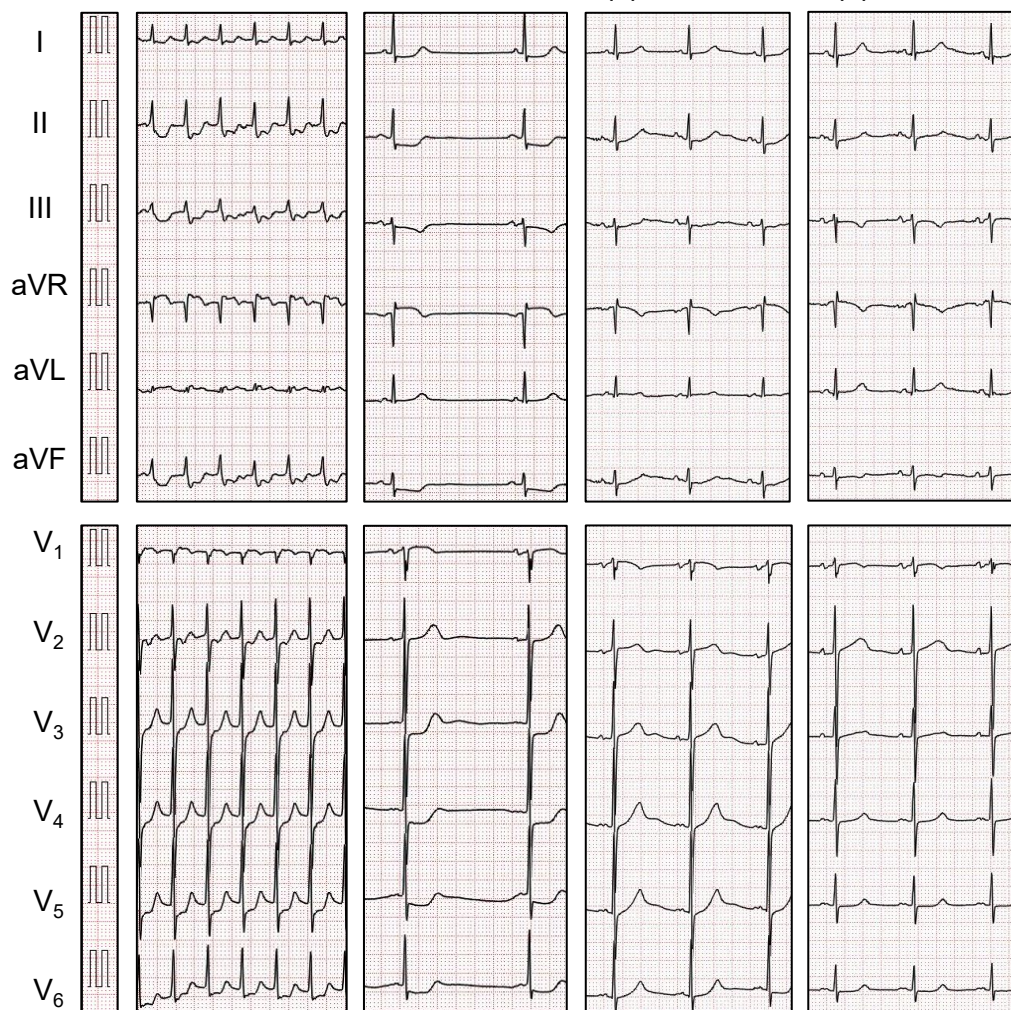

Supplement: Supplementary Figure S1 — Time-course of electrocardiographic change. Electrocardiography (ECG) on emergency arrival (A), after intravenous adenosine triphosphate (ATP) administration (B), before the ablation (ABL) (C), and after the ABL (D). The episode was a narrow QRS with short RP tachycardia. No ST-T changes were observed before or after the ABL. The ECG paper was set to a standard speed of 25 mm/s and voltage (amplitude) of 10 mm/mV. [file Datasheet1.pdf]

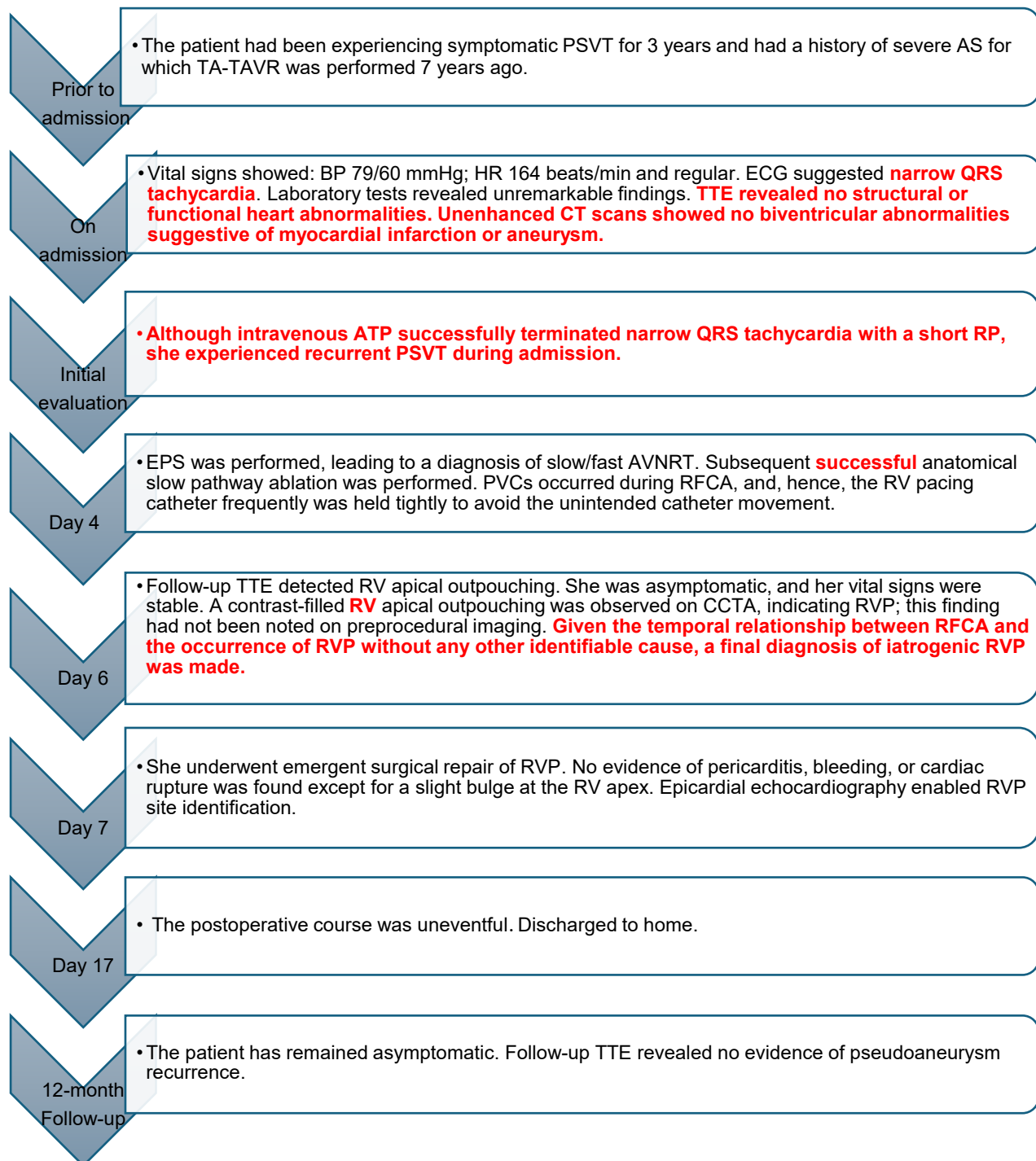

Supplement: Supplementary Figure S2 — Timeline of the diagnostics, therapeutic interventions, and disease status of the present case. AS, aortic valve stenosis; ATP, adenosine triphosphate; AVNRT, atrioventricular nodal reentrant tachycardia; BP, blood pressure; CCTA, coronary computer tomography angiography; CT, computer tomography; ECG, electrocardiography; EF, ejection fraction; EPS, electrophysiological study; HR, heart rate; PVC, premature ventricular contraction; PSVT, paroxysmal supraventricular tachycardia; RFCA, radiofrequency catheter ablation; RV, right ventricular; RVP, right ventricular pseudoaneurysm; TA-TAVR, transapical transcatheter aortic valve replacement; and TTE, transthoracic echocardiography. [file Datasheet2.pdf]
